# Efficacy and safety of the proposed bevacizumab biosimilar BAT1706 compared with reference bevacizumab in patients with advanced nonsquamous non‐small cell lung cancer: A randomized, double‐blind, phase III study

**DOI:** 10.1002/cam4.6664

**Published:** 2023-11-07

**Authors:** Likun Chen, Jose David Gomez Rangel, Timucin Cil, Xingya Li, Irfan Cicin, Yihong Shen, Zhihua Liu, Ozgur Ozyilkan, Bondarenko Igor, Jun Chen, Kostiuk Oleksandr, Zhendong Chen, Helong Zhang, Ziyi Fu, Qingfeng Dong, Shuqiang Song, Jin‐Chen Yu, Li Zhang, Adamchuk Hryhoriy, Aziz Karaoglu, Bardakov Hryhoriy, Beili Gao, Berna Oksuzoglu, Bondarenko Igor, Cemil Bilir, Christa Jordaan, Chunhong Hu, Conrad Jacobs, Deniz Tural, Dilek Erdem, Dmytro Trukhin, Dongning Huang, Feng Ye, Francisco Alejo Medina Soto, Galaychuk Igor, Garth Davids, Grygorii Ursol, Hailong Liu, Hakan Harputluoglu, Hasan Senol Coskun, Havva Yesil Cinkir, Helong Zhang, Hu Ma, Hui Zhao, Huili Zhu, Irfan Cicin, Iryna Sokur, Ivan Sinielnikov, Ivashchuk Oleksandr, Jian Fang, Jiandong Tong, Jianhua Shi, Jiazina Tuohayi, Jie Zhang, Jinghua Gao, Jiyong Peng, Jose David Gomez Rangel, Jose Luis Martinez Lira, Jun Chen, Junguo Lu, Juntao Yao, Kobziev Oleh, Kostiuk Oleksandr, Kryzhanivska Anna, Lei Chen, Li Cai, Li Zhang, Liangming Zhang, Likun Chen, Liubov Syvak, Liyan Jiang, Louis Dupper, Lydia Dreosti, Mahmut Gumus, Mehmet Artac, Muhammet Kaplan, Mustafa Erman, Natalya Lisovska, Oleksii Kolesnik, Oleksiienko Alona, Omer Olmez, Ozden Altundag, Ozgur Ozyilkan, Ozgur Tanriverdi, Ozlem Yersal Oltulu, Ponomarova Olga, Qi Li, Qitao Yu, Rene Lazaro Gonzalez Mendoza, Rusyn Andriy, Senming Wang, Shen Xu, Tao Shou, Tienan Yi, Timucin Cil, Tuncay Goksel, Wei Li, Weidong Guo, Weiqing Zhao, Wenxiu Yao, Xingya Li, Yanqiu Zhao, Yaroslav Shparyk, Yevhen Hotko, Yihong Shen, Yiping Zhang, Zhendong Chen, Zhendong Zheng, Zhengdong Wu, Zhihua Liu, Ziping Wang

**Affiliations:** ^1^ Department of Medical Oncology State Key Laboratory of Oncology in South China Guangdong Provincial Clinical Research Center for Cancer Sun Yat‐Sen University Cancer Center Guangdong Guangzhou China; ^2^ Clinical Medical Research S.C Veracruz México; ^3^ Health and Science University, Adana City Education and Research Hospital Adana Turkey; ^4^ The First Affiliated Hospital of Zhengzhou University Henan Zhengzhou China; ^5^ Trakya University Medical Faculty Edirne Turkey; ^6^ The First Affiliated Hospital of Zhejiang University School of Medicine Zhejiang Hangzhou China; ^7^ Jiangxi Cancer Hospital Jiangxi Nanchang China; ^8^ Baskent University Adana Application and Research Center Adana Turkey; ^9^ CNE “City Clinical Hospital No4” Dnipro Ukraine; ^10^ Tianjin Medical University General Hospital Tianjin China; ^11^ Podilskyi Regional Oncological Center Vinnytsia Ukraine; ^12^ The Second Hospital of Anhui Medical University Anhui Hefei China; ^13^ Tangdu Hospital, Fourth Military Medical University Shanxi Xi'an China; ^14^ Bio‐Thera Solutions, Ltd. Guangdong Guangzhou China

**Keywords:** bevacizumab, biosimilar, efficacy, nonsquamous non‐small cell lung cancer, safety

## Abstract

**Background:**

BAT1706 is a proposed biosimilar of bevacizumab (Avastin®). We aimed to compare the efficacy and safety of BAT1706 with that of EU‐sourced reference bevacizumab (EU‐bevacizumab) in patients with advanced nonsquamous non‐small cell lung cancer (NSCLC).

**Methods:**

Patients were randomized 1:1 to BAT1706 plus paclitaxel and carboplatin (BAT1706 arm) or EU‐bevacizumab plus paclitaxel and carboplatin (EU‐bevacizumab arm) given every 3 weeks for six cycles, followed by maintenance therapy with BAT1706 or EU‐bevacizumab. The primary endpoint was overall response rate at week 18 (ORR_18_). Clinical equivalence was demonstrated if the 90% confidence interval (CI) of the BAT1706:EU‐bevacizumab ORR_18_ risk ratio was contained within the predefined equivalence margins of 0.75–1.33 (China National Medical Products Administration requirements), or 0.73–1.36 (US Food and Drug Administration), or if the 95% CI of the ORR_18_ risk difference between treatments was contained within the predefined equivalence margin of −0.12 to 0.15 (EMA requirements).

**Results:**

In total, 649 randomized patients (BAT1706, *n* = 325; EU‐bevacizumab, *n* = 324) received at least one cycle of combination treatment. The ORR_18_ was comparable between the BAT1706 and EU‐bevacizumab arms (48.0% and 44.5%, respectively). The ORR_18_ risk ratio of 1.08 (90% CI: 0.94–1.24) and the ORR_18_ risk difference of 0.03 (95% CI: −0.04 to 0.11) were within the predefined equivalence margins, demonstrating the biosimilarity of BAT1706 and EU‐bevacizumab. The safety profile of BAT1706 was consistent with that of EU‐bevacizumab and no new safety signals were observed.

**Conclusion:**

In patients with advanced nonsquamous NSCLC, BAT1706 demonstrated clinical equivalence to EU‐bevacizumab in terms of efficacy, safety, pharmacokinetics, and immunogenicity.

## 1 INTRODUCTION

Lung cancer is the most common cause of cancer‐related deaths worldwide.^1^, ^2^ An estimated 2.2 million new lung cancer cases and 1.7 million lung cancer‐associated mortalities were reported in the GLOBOCAN 2020 database.^2^ In China, lung cancer is responsible for over 20% of all cancer deaths, and the number of new lung cancer cases in China in 2020 was estimated at 815,563.^3^ Non‐small cell lung cancer (NSCLC) accounts for the majority (80%–90%) of lung cancers.^4^ The 5‐year overall survival (OS) rate for NSCLC is ∼15% overall, and ∼4.5% for metastatic disease.^5^ Targeted therapies have demonstrated efficacy in patients with NSCLC and confirmed mutations in epidermal growth factor receptor (EGFR) or anaplastic lymphoma kinase (ALK) genes.^6^, ^7^ However, in patients with NSCLC without confirmed mutations in these genes, or with a mutation but no access to targeted therapy, platinum‐based chemotherapy is recommended.^4^, ^8^ Alternative, affordable treatments are needed for these patients.

Vascular endothelial growth factor (VEGF) is the central mediator of angiogenesis, the development of new blood vessels, a process that is vital for tumor cell growth.^9^ VEGF‐signaling can also support tumor progression by promoting cancer cell proliferation and metastasis.^10^ Bevacizumab is a humanized monoclonal antibody that elicits antiangiogenic effects through binding to VEGF and preventing its interaction with VEGF receptors.^10^ Bevacizumab prevents the formation of new tumor blood vessels and promotes the regression of existing tumor vasculature, and has demonstrated clinical benefit across a range of solid tumor types.^10^, ^11^ In 2006, the United States Food and Drug Administration (FDA), followed by the European Medicines Agency (EMA) in 2007, approved the use of bevacizumab (Avastin®) combined with paclitaxel and carboplatin as a first‐line treatment for late‐stage NSCLC.^10^, ^11^ In 2015, the China National Medical Products Administration (NMPA) also approved bevacizumab as a first‐line treatment for late‐stage NSCLC.^12^ Approvals were based on the results of the Eastern Cooperative Oncology Group (ECOG) 4599 trial, where the addition of bevacizumab to paclitaxel and carboplatin significantly increased OS, progression‐free survival (PFS), and overall response rate (ORR) compared with paclitaxel and carboplatin alone.^11^ Bevacizumab in combination with paclitaxel and carboplatin is now recommended in clinical practice as a standard treatment option for advanced or metastatic NSCLC.^4^, ^8^

BAT1706 is a recombinant humanized anti‐VEGF monoclonal antibody developed by Bio‐Thera Solutions, Ltd. as a proposed biosimilar of bevacizumab to meet the need for alternatives to expensive biologic agents. A biosimilar is a biological product that is highly similar to, and has no clinically meaningful differences from, an existing approved reference biologic.^13^ Biosimilars have been regarded as important by the American Society of Clinical Oncology and the European Society of Medical Oncology in enhancing patient care by facilitating affordable access to anticancer therapies.^13^, ^14^

Two Phase I clinical studies conducted in New Zealand (NCT03030430) and China (BAT1706‐002‐CR) demonstrated that a single intravenous infusion of BAT1706 (1 mg/kg) was well‐tolerated in healthy participants.^15^, ^16^

In this pivotal Phase III study (NCT03329911), we compared the efficacy and safety of BAT1706 and EU‐bevacizumab, given in combination with chemotherapy, as first‐line treatment for advanced nonsquamous NSCLC, in order to evaluate the clinical equivalence of BAT1706 and reference EU‐bevacizumab.

## 2 METHODS

### 2.1 Study design and patients

This multicenter, randomized, double‐blind, Phase III, parallel, 2‐arm study (NCT03329911) was designed to compare the efficacy and safety of BAT1706 plus paclitaxel and carboplatin (BAT1706 arm) vs EU‐bevacizumab plus paclitaxel and carboplatin (EU‐bevacizumab arm). Adult patients aged ≥18 years were eligible for inclusion if they had: histologically or cytologically confirmed Stage IV nonsquamous NSCLC or recurrent disease; tumors without EGFR mutations or ALK rearrangements; at least one measurable target lesion according to Response Evaluation Criteria in Solid Tumors version 1.1 (RECIST 1.1); received no prior systemic therapy for metastatic disease; an ECOG performance status of 0 or 1 and a life expectancy of more than 3 months; and adequate hematological, hepatic, and renal function. Key exclusion criteria included: a diagnosis of small cell carcinoma of the lung; known receptor tyrosine kinase 1 (ROS1) positive tumors; tumor cavitation, tumor invading into large blood vessels or close to large vessels with an increased risk of bleeding; having received prior therapy with monoclonal antibodies or small molecule inhibitors against VEGF or VEGF receptor; known brain metastasis or other central nervous system (CNS) metastasis that was either symptomatic or untreated; a significant thrombotic or hemorrhagic event ≤6 months prior to screening; the need for permanent oral anticoagulation treatment (eg, warfarin, dabigatran); history of poorly controlled hypertension; and history of active gastroduodenal ulcer or abdominal fistula within 6 months prior to screening.

### 2.2 Randomization and blinding

Patients were randomized (1:1) in a blinded manner to receive either BAT1706 plus chemotherapy or EU‐bevacizumab plus chemotherapy using the Interactive Web Response System (IWRS). Randomization was stratified by the following factors: stage of NSCLC (recurrent disease of any stage at the time of primary diagnosis, or Stage IV); sex (male or female); and ethnicity (Asian or non‐Asian). Patient numbers were assigned consecutively. Double‐blinding was maintained throughout the study; unblinding was only allowed in cases of an emergency for safety concerns.

### 2.3 Procedures

BAT1706 or EU‐bevacizumab, plus paclitaxel and carboplatin, were given every 3 weeks (21 days) for up to six cycles of combination therapy. BAT1706 (15 mg/kg) or EU‐bevacizumab (15 mg/kg) were administered intravenously prior to paclitaxel and carboplatin, with the initial dose given over 90 min; if well‐tolerated, subsequent infusions were given over 60 min. Dose adjustments were not permitted. Paclitaxel was administered intravenously over 3 h (or according to the package insert) at an initial dose of 200 mg/m^2^, after adequate premedication and before carboplatin was given. For Chinese patients (according to the local label), paclitaxel could be given at a dose of 175 mg/m^2^. The initial dose of carboplatin (area under the curve of 6 mg/mL/min) was administered intravenously in accordance with local best practices or the package insert.

For cycle 1, day 1, only BAT1706 or EU‐bevacizumab were given; on day 2, paclitaxel and carboplatin were administered. From cycle 2 (day 22) to cycle 6, BAT1706 or EU‐bevacizumab were administered first, followed by paclitaxel and then carboplatin on the same day. Any delay in chemotherapy necessitated a delay in BAT1706/EU‐bevacizumab administration, and vice versa, during the combination treatment.

After six cycles of combination therapy, all patients with complete response (CR), partial response (PR), or stable disease received maintenance therapy with BAT1706 or EU‐bevacizumab for up to 12 months after week 18. Maintenance therapy was stopped if any of the following occurred: investigator‐assessed disease progression, excessive toxicity, investigator's judgment, withdrawal of consent, loss to follow‐up, death, start of a new anticancer therapy, or study termination by the Sponsor, whichever occurred first.

After this 12‐month treatment period, patients still benefiting from treatment were able to enroll in a long‐term extension (LTE) study.

### 2.4 Endpoints and assessments

The primary endpoint was the ORR at week 18 (ORR_18_), calculated as the proportion of patients achieving a PR or a CR at week 18, as assessed by independent and blinded central imaging review (CIR) using RECIST v1.1 criteria. Patients who did not complete the ORR_18_ assessment were considered nonresponders if they had discontinued the study due to intolerance, disease progression, withdrawal of consent, loss to follow‐up, or death. Tumor assessments were carried out at weeks 6, 12, and 18, regardless of whether a treatment cycle was postponed, and were then performed every three cycles (approximately every 9 weeks [assessed within days 15 to 21 of the third cycle]). During the first 18 weeks, the maximum window for tumor assessment was 1 week. Contrast‐enhanced computed tomography or magnetic resonance imaging of the chest and abdomen (including the adrenals and liver, as well as any other areas of disease as clinically indicated) was obtained at screening and at all imaging time‐points. Brain computed tomography or magnetic resonance imaging scans, as well as bone scans or X‐rays, were performed at screening and during the treatment period, as clinically indicated. If the bone scan was positive at screening, it was repeated at least every 12 weeks. Plain X‐rays of bone lesions were used, as clinically indicated, between isotopic bone scans during regular tumor assessments. Until week 18, all imaging for tumor assessments were sent for CIR and used for the primary efficacy analysis.

Secondary efficacy endpoints included: CIR‐assessed ORR at week 6 (ORR_6_) and week 12 (ORR_12_); best ORR of confirmed responses at the end of the study; duration of response (DoR; CIR‐assessed up to week 18, investigator‐assessed after week 18); investigator‐assessed PFS and OS at 12 months; and investigator‐assessed duration of PFS and OS. At 12 months, additional comparative safety/immunogenicity data (eg, presence of anti‐drug antibodies [ADAs]), as well as other efficacy data (such as DoR, PFS, and OS rates), were collected from those patients with disease control who continued to receive BAT1706 or EU‐bevacizumab maintenance therapy.

All adverse events (AEs) and serious AEs were evaluated for assessment of safety. The National Cancer Institute Common Terminology Criteria for AE version 4.03 was used to grade the severity of treatment‐emergent AEs (TEAEs), which were defined as events that emerged during treatment (having been absent pretreatment or worsened relative to the pretreatment state) and with onset dates occurring between the first dosing day of study treatment, and until 28 days after the last dose of study treatment. Clinical laboratory results, electrocardiogram parameters, vital signs, and physical examinations were also assessed. AEs of special interest included anaphylactic reactions, arterial and venous thromboembolic events, febrile neutropenia, gastrointestinal perforations, hypertension, proteinuria, pulmonary hemorrhage, other hemorrhages, and wound healing complications. Blood samples were also collected throughout the study for pharmacokinetic (PK) analysis.

### 2.5 Statistical analyses

A total of 632 patients were planned to be enrolled in this study. The intention‐to‐treat (ITT) population included all randomized patients based on the intended treatment arm, regardless of treatment received. The per‐protocol (PP) population included patients in either treatment arm who had received either at least three cycles of their allocated treatment, or less than three cycles of treatment (due to early progression, death, or excessive toxicity), along with one tumor assessment and no major protocol deviations that could significantly impact on efficacy or safety outcomes. Patients who discontinued prior to week 18 for any reason were counted as nonresponders in the primary endpoint analysis for the ITT population. Efficacy analyses were carried out in both the ITT and PP populations. Safety analyses were carried out in the safety population, which included all randomized patients who received at least one dose of BAT1706 or EU‐bevacizumab, and who were assigned to that treatment.

For the primary endpoint, the risk ratio of and the risk difference between the treatment arms for ORR_18_ were analyzed to assess clinical equivalence. Clinical equivalence was demonstrated if the 90% confidence interval (CI) of the BAT1706:EU‐bevacizumab ORR_18_ risk ratio was entirely contained within the equivalence margins of 0.75–1.33, complying with NMPA requirements, or 0.73–1.36, complying with FDA requirements. Clinical equivalence was also demonstrated if the 95% CI (calculated using the Clopper and Pearson method^17^) of BAT1706/EU‐bevacizumab ORR_18_ risk difference was entirely contained within the equivalence margin of −0.12 to 0.15, complying with EMA requirements. Multivariate‐adjusted risk ratios and 90% CIs were calculated using a log‐binomial regression model with stratification factors. If the log‐binomial model failed to converge, Poisson regression with robust error variance was used.

For time‐to‐event variables such as PFS, OS, and DoR, Kaplan–Meier curves were calculated and displayed. A stratified Cox regression model yielded estimated hazard ratios (HRs) with 95% CIs, with treatment as the explanatory variable, and nonsquamous NSCLC stage (recurrent disease or Stage IV), sex (male or female), and ethnicity (Asian or non‐Asian) as stratification factors; an HR <1.0 favored BAT1706. *P* values were calculated using a log‐rank test stratified by NSCLC stage (recurrent disease or Stage IV), sex (male or female), and ethnicity (Asian or non‐Asian). Stratification factors were from an IWRS, and the CIs for the median were calculated according to Brookmeyer and Crowley.^18^ Secondary analyses were carried out based on up to a 12‐month exposure. For the safety analyses, analysis of TEAEs was based on data reported up to the cutoff date of November 05, 2019, at which point all patients had either completed 12 months of treatment, or had withdrawn from the study for any reason; TEAEs occurring during the LTE were not included.

Statistical analyses were carried out using SAS Version 9.4®. PK parameters were analyzed using Phoenix® WinNonlin® Version 8.0, with PK figures generated using SAS Version 9.4®.

## 3 RESULTS

### 3.1 Patient population and baseline characteristics

The first patient was enrolled on December 11, 2017 (study initiation date), and the results reported here are based on a data cutoff date of November 05, 2019, at which point all patients had either completed 12 months of treatment, or had withdrawn from the study.

**FIGURE 1:**
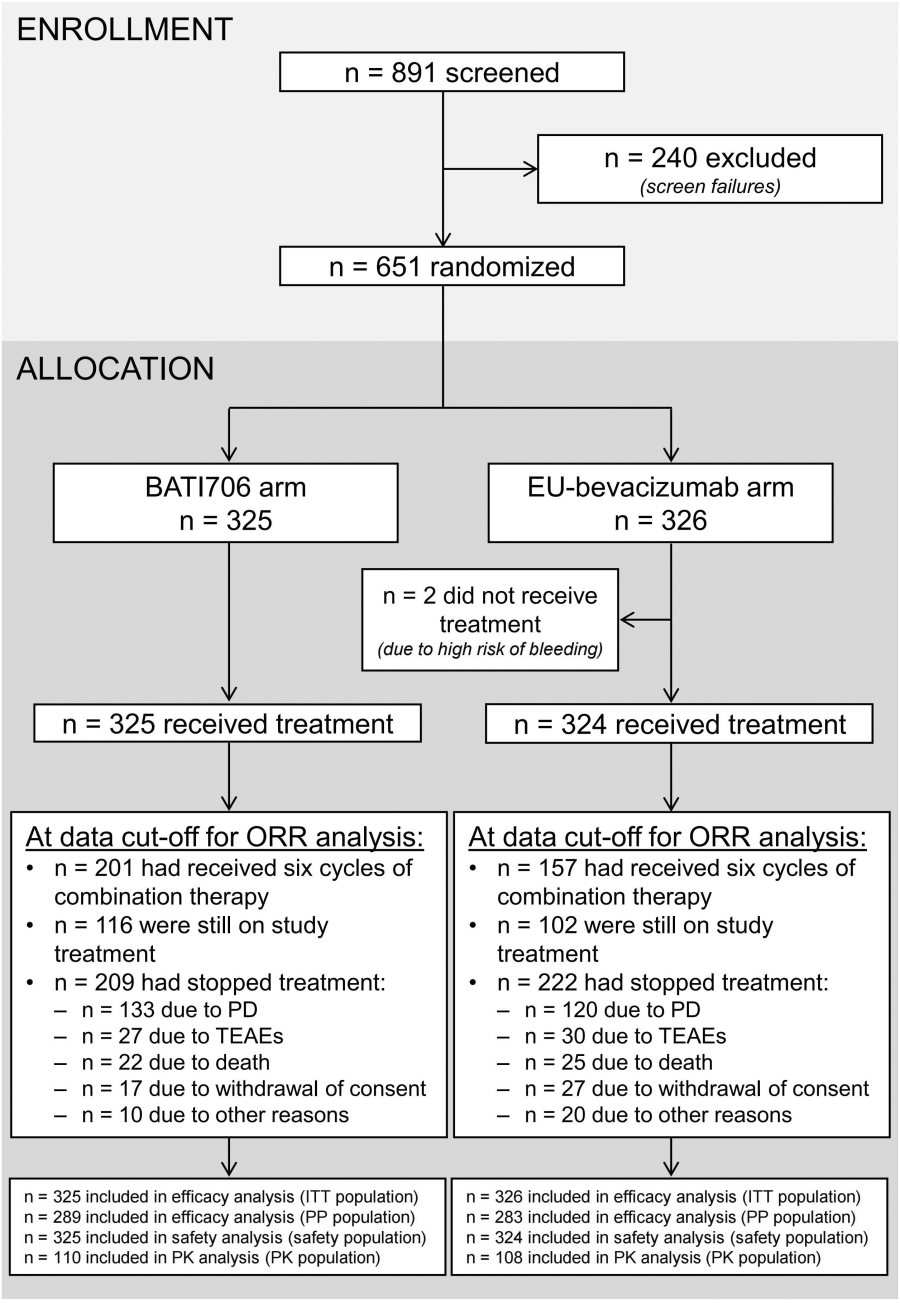
Patient disposition. ITT, intention‐to‐treat; ORR, overall response rate; PD, progressive disease; PK, pharmacokinetic; PP, per protocol; TEAE, treatment‐emergent adverse event.

In total, 651 patients were randomized (325 in the BAT1706 arm and 326 in the EU‐bevacizumab arm) and comprised the ITT population (Figure 1). Of these, two patients in the EU‐bevacizumab arm discontinued the study prior to receiving treatment, due to a high risk of bleeding. Thus, 649 patients received at least one cycle of combination treatment and were included in the safety population (325 in the BAT1706 arm and 324 in the EU‐bevacizumab arm). Overall, patient disposition was similar between treatment arms. More than half the patients (358 [55.0%]: 201 [61.8%] in the BAT1706 arm and 157 [48.2%] in the EU‐bevacizumab arm) received six cycles of combination treatment. At data cutoff, 431 (66.2%) patients had stopped treatment, with the most common reasons presented in Figure 1, while 218 (33.5%) were still receiving therapy. Of these 218 patients, 42 (6.5%) entered the LTE study: 25 (7.7%) in the BAT1706 arm and 17 (5.2%) in the EU‐bevacizumab arm.

Demographic and baseline characteristics of patients were similar for both treatment arms (Table 1). Most patients were male (70.2%) and non‐Asian (57.0%, with 52.7% white). The median age was 61 years (range: 26–88 years), median weight was 66 kg (range: 38–136 kg) and median body mass index was 24.2 kg/m^2^ (range: 15.8–45.2 kg/m^2^). In total, 581 (89.2%) patients had Stage IV cancer at the time of initial diagnosis.

**TABLE 1:** Demographic and baseline—ITT population.

	BAT1706 + Carboplatin + Paclitaxel (BAT1706 arm) *N* = 325	EU‐bevacizumab + Carboplatin + Paclitaxel (EU‐bevacizumab arm) *N* = 326	Total *N* = 651
Sex, *n* (%)			
Male	228 (70.2)	229 (70.2)	457 (70.2)
Female	97 (29.8)	97 (29.8)	194 (29.8)
Race, *n* (%)			
Asian	141 (43.4)	140 (42.9)	281 (43.2)
Non‐Asian	184 (56.6)	187 (57.4)	371 (57.0)
American Indian or Alaskan native	11 (3.4)	13 (4.0)	24 (3.7)
Black or African American	1 (0.3)	1 (0.3)	2 (0.3)
Native Hawaiian or other Pacific Islander	0 (0.0)	1 (0.3)	1 (0.2)
White	172 (52.9)	171 (52.5)	343 (52.7)
Other	0 (0.0)	1 (0.3)	1 (0.2)
Age (years)			
*n* (%)	325 (100.0)	326 (100.0)	651 (100.0)
Mean ± SD	59 ± 9.7	61 ± 9.0	60 ± 9.4
Median	60	61	61
Q1; Q3	53; 66	55; 67	54; 66
Min‐max	27–84	26–88	26–88
Age categories, *n* (%)			
<65 years	233 (71.7)	208 (63.8)	441 (67.7)
≥65 years	92 (28.3)	118 (36.2)	210 (32.3)
65– < 75 years	79 (24.3)	105 (32.2)	184 (28.3)
75– < 85 years	13 (4.0)	12 (3.7)	25 (3.8)
≥85 years	0 (0.0)	1 (0.3)	1 (0.2)
ECOG performance status, *n* (%)			
0	69 (21.2)	103 (31.6)	172 (26.4)
1	256 (78.8)	223 (68.4)	479 (73.6)
Height (cm)			
*n* (%)	325 (100.0)	326 (100.0)	651 (100.0)
Mean ± SD	166 ± 8.3	166 ± 8.9	166 ± 8.6
Median	166	167	166
Q1; Q3	161; 171	160; 172	160; 172
Min‐max	139–190	134–191	134–191
Weight (kg)			
*n* (%)	325 (100.0)	326 (100.0)	651 (100.0)
Mean ± SD	67.80 ± 14.703	68.98 ± 14.508	68.39 ± 14.606
Median	65.00	67.00	66.00
Q1; Q3	58.00; 75.00	59.00; 79.00	58.50; 77.50
Min‐max	38.00–136.00	40.00–115.20	38.00–136.00
Body mass index (kg/m^2^)			
*n* (%)	325 (100.0)	326 (100.0)	651 (100.0)
Mean ± SD	24.4 ± 4.49	24.9 ± 4.49	24.7 ± 4.49
Median	24.0	24.2	24.2
Q1; Q3	21.2; 26.8	21.6; 27.5	21.5; 27.3
Min‐max	15.8–45.2	16.0–42.6	15.8–45.2
Time since initial NSCLC diagnosis, (months)^a^			
*n* (%)	325 (100.0)	326 (100.0)	651 (100.0)
Mean ± SD	3.93 ± 9.951	4.24 ± 13.918	4.08 ± 12.093
Median	0.82	0.79	0.82
Q1; Q3	0.43; 1.84	0.33; 1.61	0.36; 1.71
Min‐max	0.03–73.07	0.00–143.80	0.00–143.80
Disease stage at time of initial diagnosis, *n* (%)			
Stage I	17 (5.2)	8 (2.5)	25 (3.8)
Stage II	8 (2.5)	8 (2.5)	16 (2.5)
Stage III	16 (4.9)	11 (3.4)	27 (4.1)
Stage IV	284 (87.4)	297 (91.1)	581 (89.2)
Missing	0 (0.0)	2 (0.6)	2 (0.3)
NSCLC pathology classification, *n* (%)			
Adenocarcinoma	315 (96.9)	310 (95.1)	625 (96.0)
Large cell carcinoma	2 (0.6)	8 (2.5)	10 (1.5)
Adenosquamous NSCLC mixed, predominant adenocarcinoma	0 (0.0)	0 (0.0)	0 (0.0)
Other	8 (2.5)	8 (2.5)	16 (2.5)
NSCLC stage at enrolment, *n* (%)			
Stage IV	303 (93.2)	305 (93.6)	608 (93.4)
Recurrent disease	22 (6.8)	21 (6.4)	43 (6.6)
Metastasis, *n* (%)			
Yes	319 (98.2)	313 (96.0)	632 (97.1)
No	6 (1.8)	13 (4.0)	19 (2.9)
Time since first metastasis, months^b^			
*n* (%)	319 (98.2)	313 (96.0)	632 (97.1)
Mean ± SD	1.22 ± 2.063	1.27 ± 2.066	1.24 ± 2.063
Median	0.66	0.79	0.72
Q1; Q3	0.23; 1.35	0.33; 1.45	0.30; 1.41
Min‐max	−0.26‐19.38	−0.13‐19.35	−0.26‐19.38
Time since first metastasis, months^c^			
*n* (%)	319 (98.2)	313 (96.0)	632 (97.1)
Mean ± SD	1.54 ± 2.072	1.60 ± 2.103	1.57 ± 2.086
Median	0.99	1.08	1.02
Q1; Q3	0.56; 1.71	0.66; 1.81	0.62; 1.74
Min‐max	0.10–19.75	0.10–20.04	0.10–20.04
EGFR mutation status, *n* (%)			
Positive	8 (2.5)	9 (2.8)	17 (2.6)
Negative	193 (59.4)	201 (61.7)	394 (60.5)
Unknown	5 (1.5)	7 (2.1)	12 (1.8)
Not done	119 (36.6)	109 (33.4)	228 (35.0)
ALK mutation status, *n* (%)			
Positive	1 (0.3)	6 (1.8)	7 (1.1)
Negative	194 (59.7)	202 (62.0)	396 (60.8)
Unknown	9 (2.8)	7 (2.1)	16 (2.5)
Not done	120 (36.9)	111 (34.0)	231 (35.5)
Missing	1 (0.3)	0 (0.0)	1 (0.2)
ROS‐1 mutation status, *n* (%)			
Positive	0 (0.0)	1 (0.3)	1 (0.2)
Negative	135 (41.5)	155 (47.5)	290 (44.5)
Unknown	69 (21.2)	58 (17.8)	127 (19.5)
Not done	120 (36.9)	111 (34.0)	231 (35.5)
Missing	1 (0.3)	1 (0.3)	2 (0.3)

*Note:* Body mass index (kg/m^2^) = weight (kg)/(height [m])^2. Percentages are based on number of patients in the population.

Abbreviations: ALK, anaplastic lymphoma kinase; ECOG, eastern cooperative oncology group; EGFR, epidermal growth factor receptor; ITT, intention‐to‐treat; min, minimum; max, maximum; NSCLC, non‐small cell lung cancer; ROS, receptor tyrosine kinase; SD, standard deviation.

^a^ Time since initial NSCLC diagnosis (months) = (date of informed consent form – date of initial NSCLC diagnosis +1)/30.4375.

^b^ Time since first metastasis (months) = (date of informed consent form – date of first metastasis +1)/30.4375.

^c^ Time since first metastasis (months) = (date of randomization – date of first metastasis +1)/30.4375.

### 3.2 Treatment exposure

At data cutoff, exposure to BAT1706 and EU‐bevacizumab was similar between the treatment arms. The median duration of therapy was 29.1 weeks (range: 3.0–62.1 weeks) in the BAT1706 arm and 27.0 weeks (range: 3.0–53.9 weeks) in the EU‐bevacizumab arm. Overall, patients in each treatment arm received a median of nine infusions of BAT1706 or EU‐bevacizumab. The median cumulative doses of BAT1706 and EU‐bevacizumab were 138.4 mg/kg and 130.1 mg/kg, respectively. Delays in BAT1706/EU‐bevacizumab dosing was reported in 161 patients in the BAT1706 arm (49.5%) and 155 patients the EU‐bevacizumab arm (47.8%), with the median dose delay being 7 days for both treatments.

Median duration of exposure to carboplatin at data cutoff was similar between the two treatment arms: 17.9 weeks (range: 3.0–25.3 weeks) in the BAT1706 arm, and 17.7 weeks (range: 3.0–27.4 weeks) in the EU‐bevacizumab arm. Likewise, the median number of carboplatin infusions was similar: six in the BAT1706 arm, and five in the EU‐bevacizumab arm. The median cumulative carboplatin dose was 3264.6 mg in the BAT1706 arm and 2992.8 mg in the EU‐bevacizumab arm. Delays in carboplatin dosing were reported by 123 (37.8%) patients in the BAT1706 arm and 117 (36.1%) patients in the EU‐bevacizumab arm, with the median dose delay being 5 days in each arm. Carboplatin dose‐modification was reported in 51 (15.7%) and 56 (17.3%) patients in the BAT1706 and EU‐bevacizumab arms, respectively. Similar results to carboplatin were found for paclitaxel exposure. Overall, there were no differences between the treatment arms in exposure to carboplatin or paclitaxel across the study.

### 3.3 Primary endpoint

Similar results were found in the ITT and PP populations for the primary endpoint; data reported here are for the ITT population. At data cutoff, all randomized patients had completed the week 18 visit or had been excluded from the disease evaluation period.

In the ITT population, the ORR_18_ was 48.0% (95% Cl: 42.5–53.6) in the BAT1706 arm and 44.5% (95% CI: 39.0–50.1) in the EU‐bevacizumab arm, as shown in Table 2. The ORR_18_ (BAT1706/EU‐bevacizumab) risk ratio was 1.08, with the 2‐sided 90% CI of 0.94–1.24 falling entirely within the equivalence margin of 0.75–1.33, in compliance with NMPA requirements, and 0.73–1.36, in compliance with FDA requirements. In addition, the multivariate‐adjusted risk ratio was 1.07, with a 90% CI of 0.93–1.22.

**TABLE 2:** Overall response rate, risk ratio and multivariate‐adjusted risk ratio, and risk difference and multivariate‐adjusted risk difference at week 18 – ITT population.

	BAT1706 + Carboplatin + Paclitaxel (BAT1706 arm) *N* = 325 (100%)	EU‐bevacizumab + Carboplatin + Paclitaxel (EU‐bevacizumab arm) *N* = 326 (100%)
Objective tumor response		
ORR_18_, *n* (%)^a^	156 (48.0)	145 (44.5)
95% CI (exact)^b^	(42.5–53.6)	(39.0–50.1)
Risk ratio (90% CI)^c^	1.08 (0.94–1.24)	
Multivariate‐adjusted risk ratio (90% CI)^d^	1.07 (0.93–1.22)	
Risk difference (95% CI)^e^	0.03 (−0.04–0.11)	
Multivariate‐adjusted risk difference (95% CI)^f^	0.03 (−0.04–0.11)	

Abbreviations: CI, confidential interval; CR, complete response; ITT, intention‐to‐treat; IWRS, interactive web response system; NSCLC, non‐small cell lung cancer; ORR_18_, overall response rate at week 18; PR, partial response.

^a^ The ORR_18_ was calculated as the proportion of patients achieving a PR or a CR at week 18.

^b^ The method of Clopper and Pearson was used to calculate CIs.

^c^ The risk ratio was estimated including covariates of stratification factors: NSCLC stage (recurrent disease after any stage at time of primary diagnosis, or Stage IV), sex (male or female), and ethnicity (Asian or non‐Asian). Stratification factors were from an IWRS system.

^d^ The multivariate‐adjusted risk ratio and the 90% CI were estimated by the log‐binomial regression model including stratification factors. Stratification factors were from an IWRS system.

^e^ The risk difference was estimated including covariates of stratification factors: NSCLC stage (recurrent disease after any stage at time of primary diagnosis, or Stage IV), sex (male or female), and ethnicity (Asian or non‐Asian). Stratification factors were from an IWRS system.

^f^ The multivariate‐adjusted risk difference and the 95% CI were estimated by the binomial regression model including stratification factors. Stratification factors were from an IWRS system.

The ORR_18_ risk difference between BAT1706 and EU‐bevacizumab was 0.03, with the 2‐sided 95% CI of −0.04 to 0.11 falling entirely within the equivalence margin of −0.12 to 0.15, in compliance with EMA requirements. In addition, the multivariate‐adjusted risk difference was 0.03, with a 95% CI of −0.04 to 0.11.

Clinical equivalence of BAT1706 and EU‐bevacizumab was demonstrated using both the risk ratio and risk difference methods, regardless of the stratification factors, and was supported by the multivariate‐adjusted risk analyses (Table 2).

### 3.4 Secondary efficacy endpoints

Similar results were found in the ITT and PP populations for all secondary efficacy endpoints; data reported here are for the ITT population.

**FIGURE 2:**
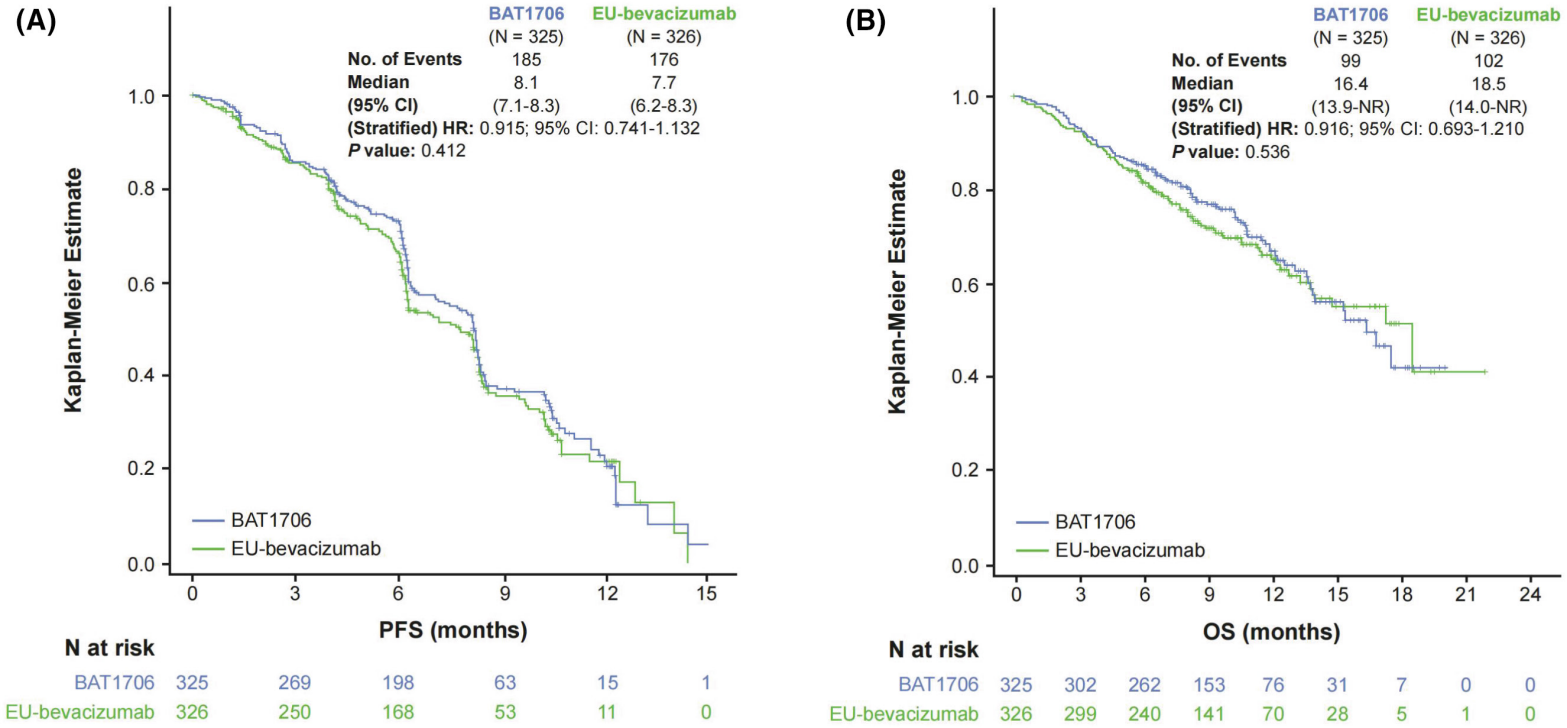
Kaplan–Meier curve of (A) PFS—ITT population and (B) overall survival—ITT population. An HR <1.0 favored the BAT1706 arm. *P* value was calculated using log‐rank test stratified by NSCLC stage (recurrent disease or Stage IV), sex (male or female), and ethnicity (Asian or non‐Asian). CI, confidence interval; HR, hazard ratio; ITT, intention‐to‐treat; NSCLC, non‐small cell lung cancer; OS, overall survival; PFS, progression‐free survival.

PFS was similar between the two treatment arms (185 [56.9%] events in the BAT1706 arm vs 176 [54.0%] event in the EU‐bevacizumab arm), with a median PFS of 8.1 (95% CI: 7.1–8.3) months for BAT1706 and 7.7 (95% CI: 6.2–8.3) months for EU‐bevacizumab, with no difference in the time to median PFS between treatment arms (stratified HR: 0.915; 95% CI: 0.741–1.132; *p* = 0.412) (Figure 2). The 12‐month PFS rate was 20.7% (95% Cl: 14.2–28.1) in the BAT1706 arm and 21.8% (95% CI: 15.0–29.5) in the EU‐bevacizumab arm.

OS was also comparable between the treatment arms (99 [30.5%] events in the BAT1706 arm vs 102 [31.3%] events in the EU‐bevacizumab arm); median OS was 16.4 (95% CI: 13.9, not reached) months for BAT1706 and 18.5 (95% CI: 14.0, not reached) months for EU‐bevacizumab, with no difference in the time to median OS between treatment arms (stratified HR: 0.916; 95% CI: 0.693–1.210; *p* = 0.536) (Figure 2). The 12‐month OS rate was also similar between treatment arms; 66.7% (95% Cl: 60.0–72.6) in the BAT1706 arm, and 65.9% (95% CI: 59.4–71.6) in the EU‐bevacizumab arm.

**FIGURE 3:**
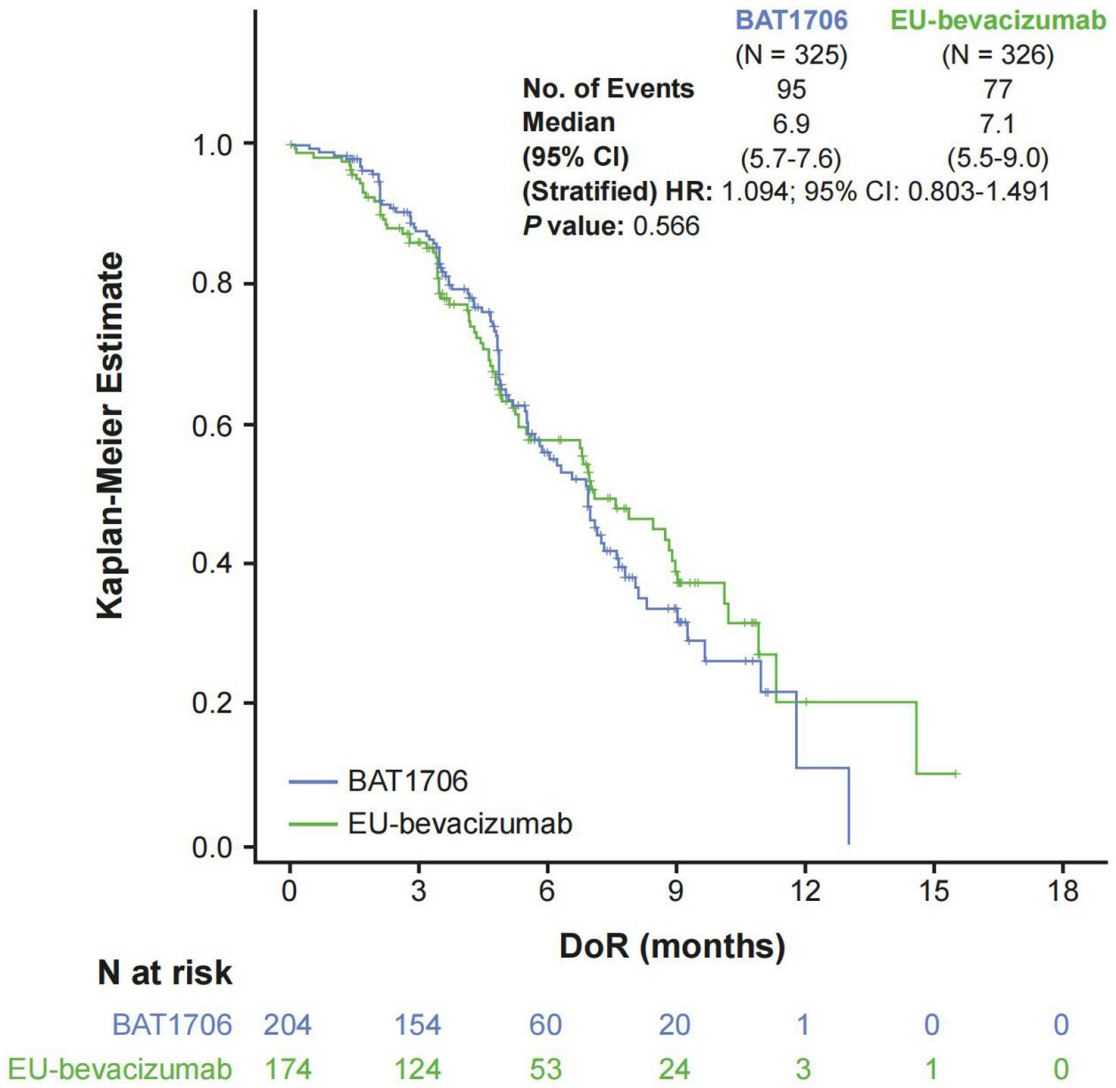
Kaplan–Meier curve of DoR—ITT population. An HR <1.0 favored the BAT1706 arm. *P* value was calculated using log‐rank test stratified by NSCLC stage (recurrent disease or Stage IV), sex (male or female), and ethnicity (Asian or non‐Asian). CI, confidence interval; DoR, duration of response; HR, hazard ratio; NSCLC, non‐small cell lung cancer.

BAT1706 and EU‐bevacizumab had comparable DoR (95 [46.6%] events in the BAT1706 arm vs. 77 [44.3%] events in the EU‐bevacizumab arm), with median DoR being 6.9 (95% CI: 5.7–7.6) months and 7.1 (95% CI: 5.5–9.0) months, respectively, with no difference in the time to DoR between treatment arms (stratified HR: 1.094; 95% CI: 0.803–1.491; *p* = 0.566) (Figure 3). The 12‐month DoR rate was 10.9% (95% Cl: 1.3–32.3) in the BAT1706 arm and 20.4% (95% CI: 8.0–36.7) in the EU‐bevacizumab arm.

In addition, ORR_6_ was 27.4% and 22.7%, and ORR_12_ was 45.2% and 40.8, in the BAT1706 and EU‐bevacizumab arms, respectively. The best overall response was 50.8% in the BAT1706 arm and 40.5% in the EU‐bevacizumab arm.

### 3.5 Safety

Overall, the incidence of TEAEs and study‐drug related TEAEs was similar between the two treatment arms. The proportion of patients who experienced at least one TEAE was 97.2% in the BAT1706 arm and 98.1% in the EU‐bevacizumab arm (Table 3); of these patients, most reported at least one BAT1706‐ or EU‐bevacizumab‐related TEAE (59.7% and 57.7%, respectively). A total of 203 (31.3%) patients reported at least one serious TEAE (98 in the BAT1706 arm and 105 in the EU‐bevacizumab arm), and of these, 32 (9.8%) and 30 (9.3%) reported at least one BAT1706‐ and EU‐bevacizumab‐related serious TEAE, respectively. In general, the two treatment arms had similar rates of TEAEs attributed to carboplatin and paclitaxel. Similar numbers of patients reported at least one carboplatin‐related serious TEAE (17.5% in the BAT1706 arm and 19.1% in the EU‐bevacizumab arm), or at least one paclitaxel‐related serious TEAE (17.5% in the BAT1706 arm and 19.1% in the EU‐bevacizumab arm).

**TABLE 3:** Summary of TEAEs of all causalities—safety population.

Number of patients with	BAT1706 + Carboplatin + Paclitaxel *N* = 325 (100%) (BAT 1706 arm) *n* (%)	EU‐bevacizumab + Carboplatin + Paclitaxel *N* = 324 (100%) (EU‐bevacizumab arm) *n* (%)	Total *N* = 649 (100%) *n* (%)
Any TEAE by preferred term in ≥5% of patients in either treatment group	316 (97.2)	318 (98.1)	634 (97.7)
Blood and lymphatic system disorders	262 (80.6)	262 (80.9)	524 (80.7)
Gastrointestinal disorders	196 (60.3)	186 (57.4)	382 (58.9)
Skin and subcutaneous tissue disorders	198 (60.9)	169 (52.2)	367 (56.5)
General disorders and administration site conditions	169 (52.0)	165 (50.9)	334 (51.5)
Nervous system disorders	160 (49.2)	159 (49.1)	319 (49.2)
Metabolism and nutrition disorders	165 (50.8)	153 (47.2)	318 (49.0)
Investigations	148 (45.5)	141 (43.5)	289 (44.5)
Musculoskeletal and connective tissue disorders	139 (42.8)	137 (42.3)	276 (42.5)
Respiratory, thoracic, and mediastinal disorders	138 (42.5)	132 (40.7)	270 (41.6)
Infections and infestations	122 (37.5)	110 (34.0)	232 (35.7)
Renal and urinary disorders	103 (31.7)	97 (29.9)	200 (30.8)
Vascular disorders	74 (22.8)	74 (22.8)	148 (22.8)
Cardiac disorders	54 (16.6)	34 (10.5)	88 (13.6)
Psychiatric disorders	42 (12.9)	33 (10.2)	75 (11.6)
Any serious TEAEs by preferred term in ≥2% of patients in either treatment group	98 (30.2)	105 (32.4)	203 (31.3)
Blood and lymphatic system disorders	31 (9.5)	46 (14.2)	77 (11.9)
Infections and infestations	27 (8.3)	22 (6.8)	49 (7.6)
Respiratory, thoracic, and mediastinal disorders	21 (6.5)	19 (5.9)	40 (6.2)
Gastrointestinal disorders	12 (3.7)	12 (3.7)	24 (3.7)
Cardiac disorders	11 (3.4)	4 (1.2)	15 (2.3)
Vascular disorders	5 (1.5)	8 (2.5)	13 (2.0)
General disorders and administration site conditions	8 (2.5)	4 (1.2)	12 (1.8)
Nervous system disorders	2 (0.6)	8 (2.5)	10 (1.5)
Metabolism and nutrition disorders	4 (1.2)	4 (1.2)	8 (1.2)
Musculoskeletal and connective tissue disorders	5 (1.5)	3 (0.9)	8 (1.2)
Renal and urinary disorders	3 (0.9)	2 (0.6)	5 (0.8)
Hepatobiliary disorders	1 (0.3)	1 (0.3)	2 (0.3)
Neoplasms benign, malignant, and unspecified (incl. cysts and polyps)	0 (0.0)	2 (0.6)	2 (0.3)
Any Grade 3 or higher TEAEs by preferred term in ≥5% of patients in either treatment	218 (67.1)	235 (72.5)	453 (69.8)
Blood and lymphatic system disorders	162 (49.8)	174 (53.7)	336 (51.8)
Neutropenia	133 (40.9)	142 (43.8)	275 (42.4)
Leukopenia	60 (18.5)	68 (21.0)	128 (19.7)
Thrombocytopenia	23 (7.1)	40 (12.3)	63 (9.7)
Anemia	20 (6.2)	27 (8.3)	47 (7.2)
Metabolism and nutrition disorders	30 (9.2)	29 (9.0)	59 (9.1)
Vascular disorders	22 (6.8)	30 (9.3)	52 (8.0)
Hypertension	20 (6.2)	23 (7.1)	43 (6.6)
Infections and infestations	29 (8.9)	21 (6.5)	50 (7.7)
Pneumonia	21 (6.5)	9 (2.8)	30 (4.6)
Investigations	16 (4.9)	34 (10.5)	50 (7.7)
Gamma‐glutamyl transferase increased	10 (3.1)	17 (5.2)	27 (4.2)
Respiratory, thoracic, and mediastinal disorders	19 (5.8)	20 (6.2)	39 (6.0)
Any Grade ≥4 TEAE	92 (28.3)	93 (28.7)	185 (28.5)
Any TEAE leading to death	18 (5.5)	17 (5.2)	35 (5.4)
Respiratory, thoracic, and mediastinal disorders	6 (1.8)	5 (1.5)	11 (1.7)
Infections and infestations	5 (1.5)	3 (0.9)	8 (1.2)
Cardiac disorders	2 (0.6)	2 (0.6)	4 (0.6)
Nervous system disorders	1 (0.3)	3 (0.9)	4 (0.6)
Gastrointestinal disorders	3 (0.9)	0 (0.0)	3 (0.5)
General disorders and administration site conditions	2 (0.6)	1 (0.3)	3 (0.5)
Blood and lymphatic system disorders	0 (0.0)	1 (0.3)	1 (0.2)
Neoplasms benign, malignant, and unspecified (incl. cysts and polyps)	0 (0.0)	1 (0.3)	1 (0.2)
Vascular disorders	0 (0.0)	1 (0.3)	1 (0.2)
Any TEAE leading to discontinuation of treatment	28 (8.6)	25 (7.7)	53 (8.2)
Respiratory, thoracic, and mediastinal disorders	8 (2.5)	5 (1.5)	13 (2.0)
Gastrointestinal disorders	7 (2.2)	4 (1.2)	11 (1.7)
Vascular disorders	3 (0.9)	6 (1.9)	9 (1.4)
Renal and urinary disorders	1 (0.3)	5 (1.5)	6 (0.9)
Infections and infestations	3 (0.9)	1 (0.3)	4 (0.6)
Nervous system disorders	2 (0.6)	2 (0.6)	4 (0.6)
Cardiac disorders	3 (0.9)	0 (0.0)	3 (0.5)
Blood and lymphatic system disorders	1 (0.3)	1 (0.3)	2 (0.3)
General disorders and administration site conditions	2 (0.6)	0 (0.0)	2 (0.3)
Eye disorders	0 (0.0)	1 (0.3)	1 (0.2)
Hepatobiliary disorders	1 (0.3)	0 (0.0)	1 (0.2)
Musculoskeletal and connective tissue disorders	1 (0.3)	0 (0.0)	1 (0.2)
Neoplasms benign, malignant, and unspecified (incl. cysts and polyps)	0 (0.0)	1 (0.3)	1 (0.2)
Skin and subcutaneous tissue disorders	0 (0.0)	1 (0.3)	1 (0.2)

Abbreviations: LTE, long‐term extension; TEAE, treatment‐emergent adverse event.

*Note*: The preferred terms in this table used US English (MedDRA version 22.1).

*Note*: National Cancer Institute Common Terminology Criteria for Adverse Events version 4.03 was used to grade the severity of adverse events.

*Note*: TEAEs were defined as events that emerged during treatment, having been absent pretreatment or worsened relative to the pretreatment state, and with onset dates occurring within the first dosing day of study treatment (BAT1706, EU‐Avastin, Carboplatin, or Paclitaxel) until 28 days after the last dose of study treatment (BAT1706, EU‐Avastin, Carboplatin, or Paclitaxel).

*Note*: TEAEs which start during the LTE were not included.

*Note*: If a patient had more than one TEAE that coded to the same preferred term, the patient was counted only once for that preferred term. Similarly, if a patient had more than one TEAE within a system organ class, the patient was counted only once in that system organ class. Patients who experienced the same coded event more than once were counted at the maximum severity.

TEAEs of Grade ≥3 were reported by 218 (67.1%) patients in the BAT1706 arm and 235 (72.5%) in the EU‐bevacizumab arm; TEAEs of Grade ≥4 were reported by 92 (28.3%) patients in in the BAT1706 arm and 93 (28.7%) in the EU‐bevacizumab arm. In total, 35 (5.4%) patients died due to a TEAE (18 patients in the BAT1706 arm and 17 in the EU‐bevacizumab arm). Among these, 11 (1.7%) patients died due to a TEAE that was considered by the investigator to be related to BAT1706 (eight patients) or EU‐bevacizumab (three patients), and a similar number of patients (12 patients; 1.8%) died due to a TEAE that was considered by the investigator to be related to carboplatin or paclitaxel. TEAEs that led to discontinuation of treatment were reported by 53 (8.2%) patients (28 patients in the BAT1706 arm and 25 in the EU‐bevacizumab arm). Discontinuations considered by investigators to be related to BAT1706/EU‐bevacizumab and paclitaxel/carboplatin were similar in both arms.

### 3.6 Pharmacokinetics

Mean serum concentrations were comparable between BAT1706 and EU‐bevacizumab throughout the study, both pre‐ and post‐dose (data not shown). For both treatments, individual serum concentrations remained quantifiable throughout the sampling interval, with mean trough concentrations comparable between BAT1706 and EU‐bevacizumab (data not shown).

### 3.7 Immunogenicity

Overall, the incidence of positive ADA results was low (≤5%) and decreased over time in both treatment arms. A total of 22 patients (12 patients in the BAT1706 arm and 10 in the EU‐bevacizumab arm), had negative ADA results at baseline and positive ADA results at the post‐baseline visit. No patients had neutralizing ADAs detected during the study. Overall, similar immunogenicity results were observed in the two treatment arms.

## 4 DISCUSSION

This Phase III biosimilar clinical study was conducted to compare efficacy, safety, PK, and immunogenicity of BAT1706 and EU‐bevacizumab in patients diagnosed with advanced nonsquamous NSCLC. Demographic and baseline characteristics were well‐balanced between the two treatment arms. Based upon equivalence margin criteria for biosimilarity stipulated by three international regulatory agencies (FDA, NMPA, and EMA), this study demonstrated the biosimilarity of BAT1706 and EU‐bevacizumab. The equivalence between BAT1706 and EU‐bevacizumab in terms of ORR_18_ was demonstrated, as the 90% Cl for the ORR_18_ risk ratio (BAT1706/EU‐bevacizumab) was entirely contained within the prespecified equivalence margins stipulated by the NMPA and FDA. In addition, the 95% CI for the risk difference in ORR_18_ between BAT1706 and EU‐bevacizumab was entirely contained within the equivalence margin stipulated by the EMA. These equivalence analyses, along with similar results found in the ITT and PP populations for ORR_18_, support the claim of clinical biosimilarity between BAT1706 and EU‐bevacizumab. Furthermore, clinical equivalence of BAT1706 and EU‐bevacizumab was shown regardless of stratification factors, as demonstrated in a multivariate‐adjusted analysis in both the ITT and PP populations. Multivariate analysis of ORR_18_ in the ITT and PP populations showed consistent results regardless of stratification factors (NSCLC stage, sex and ethnicity). Results for the secondary endpoints were consistent with those from the primary endpoint, in both the ITT and PP populations. For example, in the ITT population, median time to PFS and 12‐month PFS were similar between the two treatment arms, and notably, there was no meaningful difference in the number of patients with PFS‐related events (experienced by 56.9% and 54.0% of patients in the BAT1706 and EU‐bevacizumab arms, respectively). Overall, the BAT1706 and EU‐bevacizumab efficacy outcomes of this Phase III study are consistent with previously published Phase II and III data for bevacizumab used in combination with carboplatin and paclitaxel in treating patients with advanced NSCLC.^11^, ^19^, ^20^, ^21^

There were no notable differences between the two treatment arms regarding the incidence and severity of TEAEs and serious TEAEs, as well as changes in other safety parameters (laboratory, vital signs, and electrocardiogram). There were similar rates of BAT1706‐ and EU‐bevacizumab‐related TEAEs, along with comparable carboplatin‐ and paclitaxel‐related TEAEs, in the two treatment arms. Incidence rates of serious, Grade ≥3, and Grade ≥4 drug‐related TEAEs were also similar between treatment groups. PK and immunogenicity were also similar in the two treatment arms, with serum concentrations and exposure parameters comparable between BAT1706 and EU‐bevacizumab. Overall, the safety profile of BAT1706 was consistent with the known safety profile of reference bevacizumab published in the literature,^11^, ^19^, ^20^, ^21^ and no new safety signals or recognizable trends were observed in either treatment arm.

It is worth mentioning that the use of bevacizumab plus platinum‐based chemotherapy (paclitaxel and carboplatin) is recommended in clinical practice as a standard treatment option for advanced or metastatic NSCLC.^4^, ^8^ This recommendation was supported by a Phase III study that provided evidence on the prolongation of PFS and OS in Chinese patients with NSCLC when bevacizumab was added to platinum‐based chemotherapy.^20^ The introduction of immune‐checkpoint inhibitors (ICIs) in NSCLC has opened the opportunity to combine these agents with other targeted therapies. For example, the Phase III IMpower150 study reported that the addition of atezolizumab to bevacizumab plus chemotherapy significantly improved both PFS and OS in patients with metastatic NSCLC, with a 38% decrease in progression risk (HR: 0.62; *p* < 0.001).^22^ This combination has also shown similar benefits in hepatocellular and advanced renal carcinoma.^23^, ^24^

The goal of clinical trials on biosimilars is to demonstrate the totality of evidence on clinical equivalence,^25^ not noninferiority, and so the choice of primary objectives in such studies is of great importance. To satisfy the different regulatory agencies, several endpoints and methods of calculating equivalence were used in this global study, with predefined equivalence margins and statistical approaches used to comply with NMPA, FDA, and EMA requirements. The calculation of these equivalence margins was based on a meta‐analysis that included five randomized studies demonstrating the benefit of the addition of bevacizumab over chemotherapy alone in a similar patient population as were included in this current study.^11^, ^19^, ^20^, ^21^, ^26^ As such, our study has assiduously shown the bioequivalence of BAT1706 with EU‐bevacizumab in patients diagnosed with advanced nonsquamous NSCLC.

This study does have some potential limitations. Firstly, OS and PFS were not assessed as primary endpoints; however, it is important to note that this was a pivotal biosimilar clinical study designed to evaluate the clinical equivalence of BAT1706 and reference EU‐bevacizumab, and these endpoints are not sensitive for such an evaluation. Instead, the ORR_18_ assessed by CIR was selected as the primary efficacy endpoint, following the guidelines on biosimilar development set out by the FDA, EMA and NMPA. PFS and OS were assessed as secondary endpoints, with no significant difference found between the treatment arms in time to median PFS or OS. Secondly, after 18 weeks of study treatment, tumor assessments were performed by investigators only without central review. There may be some variation between investigators in this evaluation, but the randomized, double‐blind design of the study will minimize any variations between groups. Finally, ICIs play an important role in cancer treatment in current clinical practice, and the addition of ICIs to bevacizumab plus chemotherapy has been shown to significantly improve outcomes in patients with NSCLC. This combination regimen was not recommended as a standard treatment option in NSCLC when this study was initiated, and so ICIs were not included in the evaluated treatment regimen. The results of this study demonstrate that BAT1706 shows clinical equivalence to EU‐bevacizumab and add to the totality of evidence that BAT1706 is similar to reference bevacizumab. If BAT1706 receives regulatory approval as a bevacizumab biosimilar, physicians can be confident that BAT1706 will demonstrate clinical equivalence to reference bevacizumab in any approved combination regimen.

## 5 CONCLUSIONS

This Phase III study has demonstrated that in patients with advanced nonsquamous NSCLC, BAT1706 shows clinical equivalence to EU‐bevacizumab in terms of efficacy, safety, PK, and immunogenicity, when administrated in combination with paclitaxel and carboplatin. These data add to the totality of evidence that BAT1706 is similar to reference bevacizumab.

## AUTHOR CONTRIBUTIONS

**Li‐kun Chen:** Conceptualization (equal); formal analysis (equal); investigation (equal); resources (lead); supervision (equal); writing – review and editing (equal). **Jose David Gomez Rangel:** Data curation (equal); investigation (equal); resources (equal); writing – review and editing (equal). **Timucin Cil:** Data curation (equal); investigation (equal); resources (equal); writing – review and editing (equal). **Xingya Li:** Resources (equal). **Irfan Cicin:** Resources (equal). **Yihong Shen:** Data curation (equal); investigation (equal); resources (equal); writing – review and editing (equal). **Zhihua Liu:** Data curation (equal); resources (equal); writing – review and editing (equal). **Ozgur Ozyilkan:** Resources (equal). **Jun Chen:** Resources (equal). **Zhendong Chen:** Resources (equal). **Helong Zhang:** Resources (equal). **Ziyi Fu:** Formal analysis (equal); investigation (equal); supervision (equal); writing – original draft (lead); writing – review and editing (equal). **Qingfeng Dong:** Formal analysis (lead); writing – review and editing (supporting). **Shuqiang Song:** Conceptualization (equal); supervision (equal); writing – review and editing (supporting). **Jin‐Chen Yu:** Conceptualization (supporting); supervision (equal); writing – review and editing (equal). **Li Zhang:** Conceptualization (lead); data curation (equal); formal analysis (equal); investigation (equal); methodology (equal); writing – original draft (equal); writing – review and editing (lead). **Bondarenko Igor:** Data curation (equal); writing – review and editing (equal).

## CONFLICT OF INTEREST STATEMENT

Ziyi Fu, Qingfeng Dong, Shuqiang Song and Jin‐Chen Yu are employees at Bio‐Thera Solution Ltd. All the other authors declared that they have no conflicts of interest.

## ETHICS STATEMENT

This study was approved by an Independent Ethics Committee or Institutional Review Board. Each participating patient provided informed consent. This study (including the informed consent process) was conducted according to the ethical principles stated in the Declaration of Helsinki (64th General Assembly, Fortaleza, Brazil, October 2013), applicable International Council for Harmonization (ICH) Good Clinical Practice Guidelines (CPMP/ICH/135/95), and the applicable drug and data protection laws and regulations of the countries where the clinical study was conducted. Clinical trial registration number: NCT03329911.

## Data Availability

Data available on request from the authors
